# Review of MrsFreqPhase methods: methods designed to estimate statistically malaria parasite multiplicity of infection, relatedness, frequency and phase

**DOI:** 10.1186/s12936-024-05119-2

**Published:** 2024-10-15

**Authors:** Aimee R. Taylor, Eric Neubauer Vickers, Bryan Greenhouse

**Affiliations:** 1 Institut Pasteur, Université Paris Cité, Paris, France, Paris, France; 2 Harvard T.H. Chan School of Public Health, Boston, USA; 3 University of California San Francisco, San Francisco, USA

**Keywords:** *Plasmodium*, Genetic analysis, Analysis software, Multiplicity of infection (MOI), Complexity of infection (COI), Relatedness, Identity-by-descent (IBD), Phase reconstruction, Haplotype inference, Mixture deconvolution

## Abstract

Malaria parasites are haploid within humans, but infections often contain genetically distinct groups of clonal parasites. When the per-infection number of genetically distinct clones (i.e., the multiplicity of infection, MOI) exceeds one, and per-infection genetic data are generated in bulk, important information are obfuscated. For example, the MOI, the phases of the haploid genotypes of genetically distinct clones (i.e., how the alleles concatenate into sequences), and their frequencies. This complicates many downstream analyses, including relatedness estimation. MOIs, parasite sequences, their frequencies, and degrees of relatedness are used ubiquitously in malaria studies: for example, to monitor anti-malarial drug resistance and to track changes in transmission. In this article, MrsFreqPhase methods designed to estimate statistically malaria parasite MOI, relatedness, frequency and phase are reviewed. An overview, a historical account of the literature, and a statistical description of contemporary software is provided for each method class. The article ends with a look towards future method development, needed to make best use of new data types generated by cutting-edge malaria studies reliant on MrsFreqPhase methods.

**Supplementary Information:**

The online version contains supplementary material available at 10.1186/s12936-024-05119-2.

## Background

**Fig. 1:**
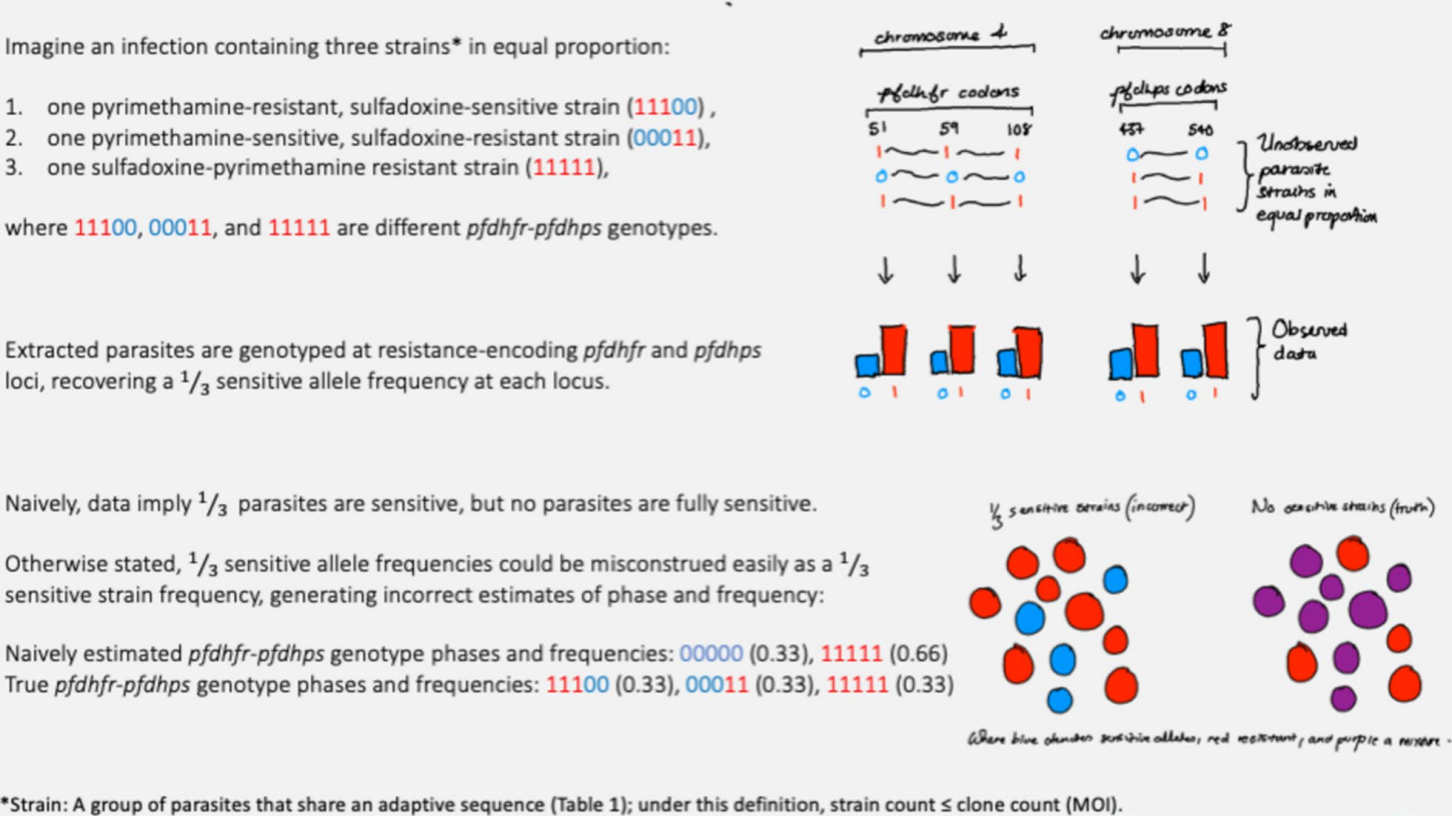
Naive phase and frequency estimation from bulk genotyping data generates incorrect estimates for a polyclonal malaria infection

Malaria parasite infections commonly contain genetically distinct groups of clonal parasites [1]. Henceforth, the word clone is used to refer to a group of clonal parasites, i.e., to a group of parasites that are genetically identical, give or take de novo mutations (see Table 1 for a list of definitions used within this review). Due to the polyclonal nature of malaria parasite infections and the bulk nature of most genotyping methods, statistical genetic methods are essential for the accurate estimation of everything from the prevalence of genetic markers of anti-malarial drug resistance to details of malaria parasite transmission. For example, to estimate the probability of a person getting infected with a malaria parasite whose genotype encodes a sequence of alleles associated with drug resistance; e.g., the *Plasmodium falciparum* quintuple *pfdhfr*-*pfdhps* mutant associated with resistance to sulfadoxine-pyrimethamine [2], a plan might proceed as follows: (1) sample parasites from infected people; (2) genotype the parasites at resistance-encoding loci; (3) concatenate per-locus data into multi-locus sequences (i.e., estimate phase); (4) estimate *θ*, the frequency of the resistance-encoding sequence among genetically distinct clones distributed across infections; (5) estimate λ, the average per-infection clone count (i.e., the population-average multiplicity of infection, MOI); (6) compute 1- (1 − θ)^λ^, the probability of being infected with one or more clones that carry the resistance-encoding sequence, under the assumption that genetically distinct clones are drawn independently; (7) assess the validity of the independence assumption by estimating relatedness between genetically distinct clones within and between infected people. Because of the polyclonal nature of malaria infections and the bulk nature of most genotyping methods (steps 1 and 2), phase, frequency and MOI estimation (steps 3 to 5) require joint statistical inference (e.g., Fig. 1). Relatedness estimation (step 7) requires statistical inference because relatedness is based on identity-by-descent (IBD), which is a hidden state.

**Table 1:** List of working definitions (some definitions vary across the literature)

Brood	A collection of parasites that hatch in unison from one or more oocysts within a mosquito
Categorical versus quantitative per-locus data	Categorical data signal the presence or absence of detection of each allele at each locus, or if the per-locus call is homo or heteroallelic. Quantitative per-locus data include read counts or other signal intensities from which within-sample allele frequencies (WSAFs) can be computed
Clone	A group of parasites that are genetically identical, give or take de novo mutations. Each clone represents a unique realization of the parasite genome
Clone proportion	Fraction of parasites belonging to a given clone within an infection
Genotype and haplotype	In this manuscript, genotype refers to an allelic sequence over loci on one or more chromosomes of the haploid parasite genome (whole-genome sequence and subsets thereof). Haplotype refers to an allelic sequence over loci on a single chromosome. For a single chromosome, haplotype and genotype are interchangeable
Heteroallelic	Loci where different alleles are detected among a collection of parasites (e.g., in a single blood sample) are referred to as heteroallelic, not heterozygous, because the signal represents a collection of genetically distinct haploid parasites, not a zygote
IBD and IBS	Identity-by-descent (identity due to common ancestry) and identity-by-state (identity due to common ancestry or chance), respectively
Microhaplotype	An allelic sequence over proximal loci whose alleles can be phased experimentally, i.e., sequenced in a single read
MOI (or COI)	Multiplicity (or complexity) of infection: the number of genetically distinct clones within an infection
Phase	How individual alleles concatenate to form a haploid sequence (haplotype or genotype)
Population	Unless specified otherwise, population is used to refer to a collection of parasites distributed across many infected humans or mosquitoes. Intra-host population refers to a population of parasites within an infection
Prevalence and frequency	Prevalence: fraction of infections that contain parasites characterised by a given allele or sequence. Frequency: relative population abundance; i.e., fraction of genetically distinct clones (or parasites) distributed within (or across) infections characterized by a given allele (or sequence). When frequencies are estimated using categorical data, they are typically fractions of genetically distinct clones. When frequencies are estimated using read count data, they are typically fractions of parasites
Strain	A group of parasites that is characterized functionally; e.g., a drug resistant or vaccine strain; elsewhere, strain is sometimes synonymous with clone
WSAF	Within-sample allele frequencies; i.e., in a sample of parasites drawn from an infection, the fraction of parasites characterized by a given allele

In this review, the focus is on MrsFreqPhase methods—statistical methods designed to estimate malaria parasite MOI, relatedness, Frequency and Phase—because they are often required together and have many common features. The estimation of IBD segments is mentioned briefly in relation to relatedness. Methods designed to estimate copy number variants (CNVs), compute linkage disequilibrium, identify selective sweeps, classify recurrent infections, and perform population assignment are beyond the scope of this review, even though some may generate MrsFreqPhase estimates as by-products. In this review, the statistical infrastructure of MrsFreqPhase methods is described, commenting on the implications of various model assumptions. MrsFreqPhase methods are not evaluated computationally, all their input requirements are not listed (see links to contemporary software, Table S1), and best approaches for different simulated scenarios are not recommended (see [3–6] for examples of benchmarking). Experimental methods (those that generate data, as well as model-free methods used to estimate MOI, relatedness, frequency and phase by e.g., counting matches) are also beyond the scope of this review; see [7] for a review of the different marker types used in malaria genomic epidemiology; see [4] for a state-of-the-art example of model-free per-infection *P. falciparum* MOI estimation; see [8] for an explanation as to why counting-methods can generate biased estimates and spurious insights when population-level frequencies are estimated using categorical data; see [9] for a general review of recent advances in malaria population genomics.

### Parasite life cycle issues

Malaria-causing *Plasmodium* parasites are single-celled eukaryotes transmitted by female Anopheline mosquitoes. Besides an obligate stage of sexual reproduction in the mosquito, they are haploid and reproduce asexually, mutating at a typically eukaryotic rate.

Throughout the life cycle, parasite numbers fluctuate immensely: transmission-stage bottlenecks are followed by massive within-host expansion [10]. Despite vast parasite counts (typically 10^8^ to 10^12^ per human), the MOI is comparatively low (typically < 10 clones per human [11]). Polyclonal infections (i.e., infections with MOI > 1) are generated in humans by co-transmission of genetically distinct parasites and/or by two or more infectious mosquito bites (superinfection). That is to say, the mainstay of intra-human parasite diversity is a direct result of the transmission process, not created de novo (in contrast with viruses that evolve within hosts). Co-transmission is ubiquitous [11–13]; superinfection scales with the entomological inoculation rate. As such, estimates of polyclonal-infection prevalence and population-average MOIs can correlate roughly with transmission intensity [13–16], making them potential indicators of disease control efforts. Per-infection MOI estimates can be used to sort monoclonal and polyclonal infections for downstream analyses, and thereby mitigate model misspecification (monoclonality is a prerequisite of many downstream analyses), and to investigate associations with host features, e.g., age [17].

Malaria parasites undergo an obligate stage of meiotic recombination in the mosquito [18]. However, some parasites self (i.e., genetically identical parasites recombine). Because parasites self, parasites from different infections can have the same whole-genome sequence, i.e., belong to the same clone (e.g., see [19]). Parasites can also be characterized by shorter sequences that many clones carry. Short sequences of interest often encode an adaptive trait or are functionally relevant. For example, drug resistance can be encoded by a sequence spanning a small number of polymorphisms on one or more chromosomes (the word haplotype is used when loci are on a single chromosome and genotype otherwise; Table 1), as well as by point mutations and CNVs [see Table 1 of 2]. In polyclonal infections, phase (how alleles in a sequence concatenate) is obfuscated, unless captured experimentally (e.g., by long-range or single-cell sequencing). At a single locus, within-sample allele frequencies (WSAFs) are obfuscated, unless captured by quantitative data (e.g., read counts). Estimates of intra-infection frequency and phase are useful for studying host-parasite interactions (e.g., [20]), and could be used to study intra-host parasite interactions, e.g., immune-mediated apparent competition [21]). At the population-level, the typical goal is to estimate the frequencies of alleles and/or sequences that encode adaptive traits, as in drug-surveillance studies, e.g., [22]. The word strain is sometimes used to refer to a group of parasites that share an adaptive sequence (e.g., a drug-resistant strain), especially when the trait is encoded by a small number of loci; elsewhere, strain is sometimes synonmous with clone (e.g., in [13] strains are defined by many thousands of whole-genome loci, and strain proportions are used to estimate the MOI).

Relatedness is a measure of IBD between a pair of parasites averaged over the genome. It is at most one between clonal individuals, reducing to zero with recombination between genetically unrelated individuals [23]. Because recombination generates genetic variation over relevantly small spatiotemporal scales, relatedness estimates are useful epidemiologically. For example, they can be used to track transmission and thus evaluate disease control efforts (e.g., [24]); to identify parasite population connectivity (e.g., [25]); to distinguish imported infections from local transmission (e.g., [26, 27]), an important use case for malaria-free certification [28]; and to identify evidence of intra-host relatedness suggestive of co-transmission [11, 13, 29]. Inbreeding coefficients are measures of IBD between two or more individuals: e.g., averaged over two haploid gametes in diploid zygotes [30, 31], or averaged over haploid parasites within human infections [32]. An inverted inbreeding coefficient is an effective MOI measure [30]: a composite measure capturing the number of genetically distinct clones and how they interrelate. The effective clone count (effective MOI) is less than the actual clone count (regular MOI) when clones are interrelated (the contribution of related parasites being penalized). It increases continuously to the actual clone count when clones are unrelated. As such, effective MOIs possibly reflect superinfection better than MOIs in high transmission settings, where intra-host relatedness is likely due to co-transmission, but not in low transmission settings, where parasites from different mosquitoes are liable to be related.

The genetic epidemiology of malaria changes radically with transmission intensity and seasonality: from holoendemic settings with perennial transmission, through settings with spatially heterogeneous and seasonal transmission, to elimination settings with occasional outbreaks [33–35]. In general, when transmission intensity is low, superinfection is infrequent, polyclonal infections are rare, and clonal propagation is frequent. Moreover, parasites across infections are likely related, leading to elevated rates of inbreeding when genetically distinct parasites do recombine. When transmission intensity is high, frequent superinfection elevates the prevalence of polyclonal infections, and thus the opportunities for recombination between genetically unrelated parasites (outbreeding), suppressing both clonal propagation and parasite relatedness between infections. Intra-host relatedness and inbreeding are both viable across the many epidemiologies of malaria: regardless of transmission intensity, mosquitoes are able to co-transmit recombinant parasites from the same brood [12, 13], and subsequent human-to-mosquito co-transmission of said parasites creates an opportunity for inbreeding, even in high transmission settings [11, 12, 30, 31]. In other words, malaria parasite mating is neither clonal nor panmictic (see [36] and references therein), and varies with transmission intensity. When transmission intensity is very high, different analyses of the same data are consistent with non-negligible inbreeding [30] and panmixia [37], providing substructuring over human infections is accounted for. Observations are consistent with both inbreeding and outbreeding in high to medium transmission intensity settings [31]; with predominant inbreeding in low transmission intensity settings [38, 39], and with selfing in clonal outbreaks [40]. Meanwhile, inter-host population structure manifests on different scales; e.g., parasite subpopulations sampled from different households in a high intensity region or spatially distinct pockets of residual transmission in a near elimination setting. Different settings will thus benefit from relatedness estimation on different scales (e.g., within versus between households in a high transmission setting).

## Methods

Table 2 lists named MrsFreqPhase methods that have been published and are readily available. They have overlapping capabilities, and are constructed around a set of common building blocks (Table 3), within either a frequentist or Bayesian framework; see below and [41]. Features and assumptions of the likelihoods of the models underpinning the methods in Table 2 are described later. Unless otherwise specified, perfect detection of alleles (i.e., 100% sensitivity) is assumed.

**Table 2:**
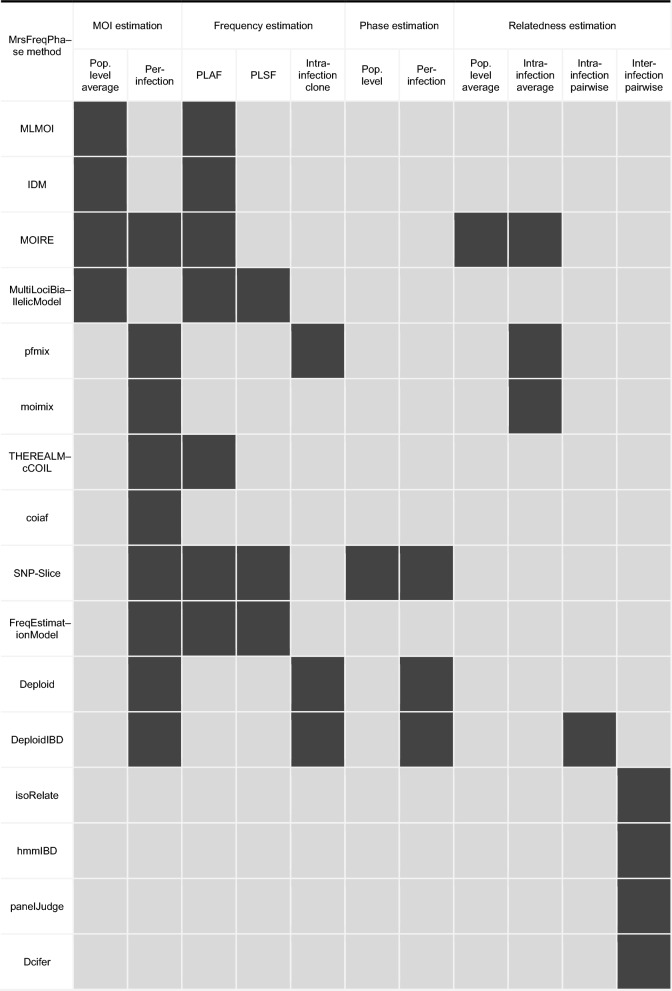
Capabilities of MrsFreqPhase software ordered by appearance

MOI (multiplicity of infection), Pop. (population), PLAF (population-level allele frequency), PLSF (population-level sequence frequency)

**Table 3:** Variables and distributions that typically feature in MrsFreqPhase methods

MOI	A Poisson or negative binomial random variable whose mean parameter is the population-average MOI. Distributions are almost always non-zero conditioned because malaria negative cases are almost always excluded from data sets analysed by MrsFreqPhase methods
Biallelic/Multiallelic locus	A Bernoulli/categorical random variable whose parameter is either a WSAF or PLAF
Sequence in PLSF estimation	A realization of a categorical random variable whose parameter is a PLAF (notable exception: SNP-Slice)
Allele frequencies	Beta random variables at biallelic loci; Dirichlet random variables at multiallelic loci
Clone proportions and PLSFs	Dirichlet random variables (notable exception: SNP-Slice)
Read count	A beta-binomial random variable if read counts are considered over-dispersed; otherwise, a binomial random variable

MOI (multiplicity of infection), PLAF (population-level allele frequency), PLSF (population-level sequence frequency), WSAF (within-sample allele frequency)

### Population versus infection-level estimation

The term population-level estimation is used to refer to the estimation of quantities averaged across parasites distributed among many infected humans or mosquitoes, and infection-level estimation to refer to the estimation of quantities at the level of an individual host.

Population-level estimation methods support relatively uninformative per-locus data that pre-date the genomic era (e.g., categorical data specifying allelic presence/absence or homo/heteroallelic calls) because they borrow information across infections. As a result, they cluster methodologically and chronologically. Infection-level estimates (e.g., per-infection MOI estimates, and estimates of intra-infection haplotype frequency and phase) can be derived from population-level estimates a posteriori [41], but they are liable to be imprecise if the per-infection data used are not very informative.

Infection-level estimation methods require relatively informative per-infection data (e.g., categorical data on many loci and/or quantitative per-locus data). Some generate population estimates by modelling data on all infections jointly, e.g., [42, 43], thereby borrowing information across infections, but also requiring many infections, which can be problematic when transmission intensity is low [4]. Population estimates generated by averaging per-infection estimates obtained separately do not leverage information borrowed across infections.

Molecular surveillance studies often centre around population-level prevalence and frequency estimation using categorical data on loci that encode adaptive traits. Meanwhile, genomic epidemiology studies often feature relatively informative per-infection data from which infection-level MOIs and population-level allele frequencies are estimated [42]. The different perspectives of molecular surveillance and genomic epidemiological studies are starting to converge around amplicon sequencing panels that combine diverse markers, including some that are neutral, with markers of drug resistance, e.g., [44, 45].

### Data types and implication

The first MrsFreqPhase methods were used to analyse data from analyses of enzymes with electrophoretically distinct variants [46, 47]. Subsequent methods have been developed around various data types including categorical data from gel-based characterization of SNPs; categorical data on microsatellites; read counts from whole-genome sequencing (WGS); and prevalence data from amplicon sequencing. Statistical methodology should adapt to different data types: e.g., by harnessing all available information (e.g., read counts from amplicon sequencing), and by customizing the observation models that capture the disconnect between latent alleles and possibly erroneous observations. For example, with sequencing methods the probability of not detecting an allele (i.e., a false negative) of a clone present at low proportion within a polyclonal infection is in part a function of amplification and sequencing methods; this probability will likely be lower for deep amplicon sequencing than WGS. The probability of detecting an allele which is not present (i.e., a false positive), is in part a function of the extent and fidelity of amplification and sequencing error rates. Such false negative and false positive error rates can be estimated to some extent by performing detailed experiments on laboratory controls, but are often not measured rigorously and even when they are can vary based on details of the specific assay, laboratory, and operator. For more examples of genotype calling artefacts and errors associated with different marker types used in malaria genomic epidemiology see [7]. Albeit not ideal, misspecification and squandered information do not render a statistical method obsolete. As such, statistical methodology lags behind experimental methodology. Experimental methods that aim to resolve within-host diversity directly (e.g., single-cell and long-read sequencing) may someday render obsolete many MrsFreqPhase methods [48]. However, these sophisticated experimental methods are not yet optimized or accessible at scale.

As alluded to above, any adequately formatted data can be fed into a statistical method at the risk of some misspecification. What goes in, comes out: if relatedness is estimated using markers under selection, for example, estimates will reflect how parasites are related at those markers. Surveillance studies for drug resistance or other important parasite phenotypes often focus on loci under selection, e.g., SNPs within the *P. falciparum* gene *kelch13* associated with artemisinin resistance [49]. As such, population-level frequency estimation methods that are designed primarily for surveillance are generally intended for data on loci under selection. Studies requiring per-infection information on within-host diversity (e.g., MOI), and/or estimation of parasite relatedness between infections (e.g., to distinguish recrudescence from reinfection in therapeutic efficacy studies, or to identify evidence of local transmission in studies of malaria-free certification), often are best served using data from highly diverse loci [42, 50, 51]. The most diverse loci in the *P. falciparum* genome tend to be regions where DNA replication error is most likely to occur (e.g., microsatellites, genes with other tandem repeats) and/or loci under balancing selection from the human immune system (e.g., *msp2*, *ama1*) [52]. Putatively neutral markers have the theoretical advantage of providing less bias than those under balancing selection with respect to relatedness estimation, but highly diverse neutral markers such as microsatellites are cumbersome to evaluate, tend to have high error rates in polyclonal infections (both false negatives and positives), and have elevated mutation rates, obfuscating estimates of IBD.

Markers under balancing selection may provide biased estimates of relatedness in theory, but some diverse targets such as microhaplotypes are amenable to accurate, high throughput genotyping and have relatively low mutation rates, providing advantages that can offset theoretical concerns [53]. Markers providing the benefit of extremely high diversity such as *msp2* unfortunately suffer from all the issues listed above: high genotyping error rates, high mutation rates, and are under balancing selection [54–56]. Thus, there are tradeoffs inherent in the choice of high diversity markers for epidemiologic studies; again, see [7] for a recent review of the different marker types used in malaria genomic epidemiology, including, antigen genes, microsatellites, SNP barcodes, *var* genes and microhaplotypes.

### Epidemiological settings and implications

MrsFreqPhase methods apply in all settings with polyclonal infections typed using standard experimental methods that generate bulk genetic data (e.g., not single-cell sequence data). In addition, relatedness estimation is useful in settings without polyclonal infections, e.g., to identify the origin of a clonal outbreak. Statistical population-level frequency estimation is particularly important when frequencies across settings with different average MOIs are compared; e.g., to know if a drug-resistant strain is selected for over time or in space, one cannot simply compare the counts of infections that test positive for the strain—the prevalence of the strain will increase dramatically with the average MOI even if its frequency remains fixed.

Settings with different average MOIs are liable to also have different relative rates of cotransmission and superinfection, and thus different relative contributions of related and unrelated parasites within polyclonal infections. When MOIs are modelled as Poisson distributed random variables, it is assumed implicitly that intra-host clones are transmitted independently; i.e., without co-transmission. Methods that model MOIs as negative binomial random variables assume implicitly that transmission events are positively correlated. As such, they are arguably better specified, especially when the relative rate of co-transmission exceeds that of superinfection. Some methods model intra-host relatedness explicitly (Table 2), whereas others do not. All polyclonal infections are liable to contain some interrelated parasites due to cotransmission, which can occur in any transmission setting where polyclonal infections exist. However, in high transmission intensity settings, where parasites across infections are generally unrelated, frequent superinfection likely elevates the relative contribution of unrelated parasites within polyclonal infections. The impact of ignoring intra-host relatedness is case specific: point estimates of population-level frequencies generated using methods that ignore intra-host relatedness should be unbiased, providing alleles/sequences distribute equally across infections with different levels of intra-host relatedness (estimates might be spuriously precise, however, because the number of independent clones from which to estimate frequencies is not as high as it seems). Meanwhile, MOIs estimated using methods that ignore intra-host relatedness are liable to be biased downwards (data on alike clones being harder to tell apart). Otherwise stated, MOI estimation ignoring within-infection relatedness is liable to generate estimates that resemble effective MOIs.

Benchmarking studies use data simulated under one model to test the performance of other models, and thus can ascertain the suitability of different methods under different simulated transmission settings. For example, [4] found MOI estimates generated by varcoding (a model-free approach based on counting distinct *var* genes) are superior to MOI estimates generated by THEREALMcCOIL when high transmission intensity is simulated under the agent-based model varmodel3 [57, 58]. The ramifications for public health depend on the use case: consider an MOI estimate interpreted as a proxy measure of the force of infection (FOI). If it is estimated ignoring intra-host relatedness, it will underestimate the true MOI across settings, and underestimate the FOI in settings where inter-host parasites are related. If it is estimated while accounting for intra-host relatedness, it will accurately estimate the true MOI across settings, but overestimate the FOI in settings where inter-host related parasites are largely unrelated and intra-host parasites are largely co-transmitted. (Ideally, FOI would be estimated directly, using a model that accounts for changing levels of inter and intra-host parasite relatedness.)

### Frequentist versus Bayesian MrsFreqPhase methods

MrsFreqPhase methods are either Bayesian or frequentist. Methods of one type or the other tend to rely on similar algorithms for inference, tend to treat missing data (e.g., NAs) similarly, tend to output the same type of point estimate, and tend to generate similar measures of uncertainty. More specifically, frequentist MrsFreqPhase methods generally generate maximum likelihood estimates (MLEs) using an optimization algorithm, e.g., the expectation–maximization algorithm [59], which can be problematic if the likelihood has local maxima. Confidence intervals can be generated using the profile-likelihood approach or asymptotic assumptions of normality (e.g., see [60, 61]), or by bootstrapping loci if they are assumed independent (e.g., see [62]). Typically, Markov-chain Monte Carlo (MCMC) samplers are used to infer parameters of Bayesian MrsFreqPhase models. MCMC samplers generate numerical approximations of posterior distributions from which posterior means or medians and credible intervals can be derived. Although less sensitive to local maxima, MCMC samplers can still get stuck, particularly if care is not taken to check for convergence. In addition, they can be computationally expensive. Regarding missing data (e.g., NAs), when infection-level estimates are generated one-by-one, loci without data can often be discarded. If so, per-infection estimates will be based on different loci counts. Otherwise, under frequentist frameworks, missing data are either imputed in a pre-inference step, imputed within the expectation–maximization algorithm, or infections with missing data are discarded, resulting in data loss. For Bayesian methods, recursive sampling within a MCMC scheme can be used to average over latent random variables, which can include missing data values. All MrsFreqPhase methods typically assume the missing mechanism is ‘ignorable’, i.e., the probability that a datum is missing does not depend on its unobserved value.

### Population-average MOI estimation

***Overview*** Population-average MOI estimation aims to directly estimate the per-infection clone count averaged across infections by jointly modelling data on many infections (in contrast to averaging per-infection MOI estimates).

***Historical context*** The first MrsFreqPhase method was designed to estimate the population-average MOI [46]. Two decades later, population-average MOI estimates were obtained indirectly from data on *P. falciparum* zygotes sampled from single-oocyst mosquitoes: firstly, under a negative binomial model of Hardy–Weinberg equilibrium with substructuring over human infections [37]; secondly, by inverting population-level inbreeding coefficients estimated from the zygote data, thereby generating effective MOI estimates [30]. Population-average MOI estimates were then estimated directly, by fitting a frequentist population-average MOI estimation model to *P. falciparum* data extracted from human infections [47]. The MOI model was designed for data on two biallelic or triallelic loci. In 2014, a single-locus version, extended to allow more than three alleles, was published, alongside statistical tests to evaluate estimates generated separately using data on different loci [60, 63]. Sometime later, bias in the single-locus model was described [61] and corrected [64]. The statistical framework of the single-locus model was extended to multiple loci in a comprehensive article on population-average MOI estimation [41]. Most recently, the single-locus model was extended to account for imperfect detection [65].

#### Contemporary software

The population-average MOI can be estimated directly using MLMOI, which is an R package built around the aforementioned single-locus, bias-corrected model [66]; and the related imperfect-detection model (see R script and documentation provided in the supplementary material of [65]). It can also be estimated using MOIRE and MultiLociBiallelicModel. MLMOI is useful if data are limited to a single locus. The imperfect-detection model (IDM) requires data on empty records: infected individuals with only missing data. Since disease-free individuals could conflate inference, IDM’s use requires independent evidence of infection positivity, or data on enough loci for evidence of infection for at least one locus. In case of multiple loci, multiple population average MOI estimates can be generated for each locus separately—see Fig. 11 of [65]. Under both MLMOI and the IDM, infections are modelled as binary vectors indicating allelic absence/presence and infection-level MOIs are modelled as random variables from a zero-truncated Poisson distribution, implying clones are transmitted independently (i.e., without co-transmission). The IDM distinguishes between observed vectors with possible false negatives, and perfectly detected latent vectors, integrating over latent vectors, assuming a fixed probability of detection. Neither MLMOI nor the IDM consider false positives (detection of absent alleles).

### Per-infection MOI estimation

***Overview*** Per-infection MOI estimation aims to estimate the number of genetically distinct clones per infection. It is typically based on either a statistical model fit to data on many loci per infection (see below); or on the per-infection maximum or near-maximum allele count observed among highly polymorphic loci (a model-free approach).

***Historical context*** Because statistical per-infection MOI estimation methods require relatively informative per-infection data, they are relatively modern. The story begins with the *F*_*WS*_ statistic [67, 68]—a composite measure of the per-infection number of genetically distinct clones, their proportions and relatedness values, which can be viewed as an inbreeding coefficient [32]. Early methods designed to estimate MOI specifically include estMOI and COIL [69, 70]. They were superseded by THEREALMcCOIL [43]. In the interim, two R packages, pfmix and moimix, both of which can be used to estimate both *F*_*WS*_ and MOI, were developed. Most recently, Coiaf [71], MOIRE [42] and SNP-Slice [3] have been added to the suite of statistical per-infection MOI estimation methods.

***Contemporary software*** Per-infection MOIs can be estimated using all the aforementioned methods. Pfmix and moimix generate MOI estimates for each infection separately using a mixture model fit to WGS read count data on biallelic SNPs, which are assumed independent [32, 72, 73]. The pfmix model is relatively elaborate. It includes intra-host clone proportions and a panmixia coefficient—some fraction of intra-host diversity that is explained by panmixia and not by the MOI—and is embedded within a Bayesian framework [72]. An MCMC sampler that splits and merges clone proportions when jumping reversibly between MOI values is described alongside the model [72], but like moimix, pfmix generates MOI estimates by model comparison [74]. MOI estimates can also be generated using methods designed to phase infection-level data. Like pfmix, moimix, and the methods designed to phase infection-level data, estMOI and COIL generate per-infection MOIs for each infection separately.

THEREALMcCOIL [43] estimates per-infection MOIs and population-level allele frequencies (PLAFs) for many infections jointly using data on many biallelic SNPs, which are assumed independent, within a Bayesian framework (each SNP requires data on at least 20 infections; each infection requires data on at least 20 SNPs). It features two methods: the categorical method for homoallelic/heteroallelic calls, and the proportional method for WSAFs derived from semi-quantitative values (THEREALMcCOIL neither requires nor explicitly supports read count data). Both methods account for genotyping errors. Under the categorical method, the observation model is a function of two miscall rates (a homo-to-het rate and het-to-homo rate—false-positive and false-negative, respectively) and the latent call; the likelihood of the latent call is computed assuming alleles are binomially distributed with probability equal to the PLAF and an MOI number of alleles, thereby implying independence between clones. Under the proportional method, the observed frequency is modelled as a Guassian random variable with mean equal to the latent WSAF and variance equal to a measurement error parameter, which is inversely proportional to signal intensity. The latent WSAF is distributed according to a mixture with point masses at 0 and 1, and a beta distribution in between. The parameters of the beta distribution are estimated pre-analysis by fitting beta distributions to WSAFs simulated for different MOI values and PLAFs. PLAFs, WSAFs, and MOIs are uniformly distributed a priori, using an MOI upper bound of 25. Error rates can also be estimated, using a uniform prior between 0 and 0.2; otherwise, they are treated as fixed.

Coiaf [71] estimates per-infection MOIs for each infection separately using data on many biallelic SNPs, which are assumed independent, within a frequentist framework. It features two methods called variant and frequency. Both methods are based on minimizing differences between observations and expectations, where differences are sums of squares that are read-depth weighted, such that SNPs with higher coverage contribute more to optimization. Differences are minimized over either a discrete or continuous domain of MOI values. Observations are either categorical homoallelic/heteroallelic calls (variant method) or WSAFs (frequency method). Expectations are functions of the MOI of interest and PLAFs, assuming alleles are binomially distributed, and thus that clones are independent (i.e., unrelated). Although coaif supports categorical data, read counts are required to weight observations by coverage, and data on WSAFs are needed to estimate PLAFs pre-analysis (PLAFs and MOIs are not estimated jointly). Both the variant and frequency method scale efficiently to thousands of SNPs and compare well with THEREALMcCOIL, especially when point estimates without confidence intervals suffice. Sequencing errors are not modelled but the software can pre-process false positives assuming loci with minor WSAFs below an error threshold are homoallelic. The error threshold (default 0.01) can be user-specified or computed internally by comparing observed and expected WSAFs.

MOIRE [42] is the most comprehensive per-infection MOI estimation method. Like THEREALMcCOIL, it estimates per-infection MOIs and PLAFs for many infections jointly using data on many loci, which are assumed independent, within a Bayesian framework. Per-infection observations are variable-length binary vectors of locus-wise allelic absence/presence, i.e., unlike THEREALMcCOIL and coiaf, MOIRE is not limited to biallelic markers. MOIRE, however, is limited to categorical data. The MOIRE model does not assume clones are unrelated: it features an average inter-clone relatedness parameter, estimates of which can be used to estimate an effective MOI that scales linearly between one and a given MOI with decreasing relatedness. The observation model is a function of two per-infection rates: a false allelic presence and absence rate, identical for all alleles. For a given locus and per-infection MOI (Poisson-distributed according to a population-average MOI), the latent-allele model is a mixture over some number of genetically distinct but related clones (binomially distributed according to the relatedness parameter) and some residual number of unrelated clones. The alleles of the unrelated clones are modelled as categorical draws from a multinomial distribution parameterized by the PLAFs (which, a priori, are uniformly distributed per-locus). The alleles of related clones are copies with probability one. Owing to a gamma hyperprior on the population-average MOI, MOIRE generates a population-average MOI estimate directly. A priori, relatedness parameters are beta distributed; error rates are capped by user-specified bounds, rescaled and then beta distributed; and PLAFs are Dirichlet-distributed with concentration parameter one.

SNP-Slice [3] is a Bayesian nonparametric method designed to reconstruct the sequences of parasites circulating in a population and estimate sequence-to-infection assignments, by jointly modelling biallelic SNP data on many infections. The per-infection sum over sequence assignments provides a per-infection MOI estimate. The MOI estimate is technically a lower bound, because sequences are counted only once per infection, whereas intra-host genetically distinct clones could share identical sequences, especially if the per-infection data are limited to few loci, to loci under selection, or to loci on a single chromosome. In this capacity—per-infection MOI estimation—SNP-Slice is presented and compared with THEREALMcCOIL in [3]. Like THEREALMcMOIL (and coiaf and MOIRE), loci are assumed independent a priori. However, unlike other methods, sequences are reconstructed within a Bayesian nonparametric framework (more details later). For each infection, the per-locus data can either be categorical (detection of the minor allele only, major allele only, or both), or minor and major read counts—benchmarking suggests read count data are preferable in all but low intensity settings [3]. Unike MOIRE, SNP-Slice does not generate estimates of relatedness. Sequence-to-infection assignments are Bernoulli trials parameterized by sequence assignment probabilities, implying inter-sequence independence. Genotyping errors are modelled using the same observation model as THEREALMcCOIL when data are categorical. Genotyping errors in read count data are accommodated insofar as read counts are modelled as random variables from either a binomial, Poisson, or negative binomial distribution.

### Population-level allele frequency estimation

***Overview*** PLAF estimation aims to estimate the frequencies of alleles distributed among clones or parasites across infections, where alleles themselves are not subject to phase ambiguity (e.g., single nucleotides, microsatellite repeats, and microhaplotype sequences that are experimentally phased by amplicon sequencing). Some PLAF estimation studies focus on alleles that confer adaptive traits (e.g., markers of antimalarial resistance); others focus on alleles at neutral loci (or loci under balancing selection) whose frequencies can be used to estimate other quantities of interest, e.g., *F*_*WS*_ [4, 67, 68]. PLAF estimation necessarily requires data on many infections. PLAF estimates can be generated by simply averaging WSAFs based on e.g., read counts (or other quantitative per-locus read outs), under the assumption that WSAFs are representative of PLAF (model-free approach). When data are categorical, PLAF estimation necessitates integration over latent intra-host clone assignments and thus statistical inference. That said, statistically unprincipled ad hoc counting is often used to estimate PLAFs from categorical data—a model-free approach that generates biased estimates and/or squandered data [8].

***Historical context*** The first MrsFreqPhase method was capable of generating PLAF estimates [46]. The first method designed specifically to target PLAFs centred around a Bayesian model of a single biallelic SNP [75]. It was followed by a suite of methods designed to estimate population-level sequence frequencies (PLSFs), but capable of generating PLAF estimates (next section). In 2017, a study was published comparing various novel frequentist methods to estimate single biallelic SNP frequencies using summary statistics from surveillance studies; i.e., counts of infections that are purely resistant, purely wild-type, or heteroallelic at the locus of interest [22]. The methods accommodate undetected clones, which undermine rare-variant frequency estimation [1, 8], under a variety of different detection mechanisms: detection due to limit of detection over clone counts; detection due to some fixed probability of detection; detection due to some fixed probability of detection that decreases linearly with MOI; and detection due to some fixed probability of detection for a dominant clone, which exceeds some smaller detection probability for all other clones. A model under which data are aggregated over multiple populations with different allele frequencies was also described. Very clear example R code was provided, but no software.

***Contemporary software*** PLAF estimates for multiallelic loci modelled separately can be generated using MLMOI and the related IDM. They can be generated for many loci modelled jointly assuming inter-locus independence using THEREALMcCOIL (biallelic loci) and MOIRE (multiallelic loci). They can also be estimated from prevalence data using methods designed to estimate PLSFs, either by fitting the models to data on individual loci separately or by summing over relevant sequence frequency estimates (Box 1).

**Table:** 

Box 1: Estimating population-level allele frequencies from population-level sequence frequencies
Imagine you want to estimate PLAFs at three biallelic loci using a method designed to estimate PLSFs.
First, using the method designed to estimate PLSFs, estimate the frequencies of sequences 000, 100, 010, 001, 110, 101, 011, and 111, where 0 denotes a reference allele (e.g., the wild-type allele) and 1 denotes the alternative allele at each of the three biallelic loci.
Second, estimate the frequency of the reference allele at a given locus by summing over the frequencies of all sequences with the reference allele at the given locus. For example, to estimate the frequency of the reference allele at the first locus, sum over frequencies for 000, 010, 001 and 011.

### Population-level sequence frequency estimation

**Overview** PLSF estimation aims to estimate the frequencies of sequences that are not phased experimentally (i.e., multi-locus haplotypes and genotypes) distributed among clones or parasites across infections, by modelling data on many infections jointly (in contrast to post-processing the output of infection-level phase and frequency estimation methods; next section). When phase is experimentally attainable (e.g., for polymorphisms within 250 base pairs using paired-end 150 base pair sequencing), PLAF estimation methods apply. Otherwise, phase ambiguity calls for more elaborate methods that integrate out latent phases. As for PLAFs, ad hoc counting methods are sometimes applied to categorical data, generating inherently biased estimates, and/or squandered data [8]. Most statistical PLSF estimation methods are limited to moderate loci counts (e.g., 8–10 SNPs at most) because they model sequences as categories, and the number of categories grows exponentially with the number of loci [41, 76].

***Historical context*** The first method capable of generating frequency estimates for sequences (over two biallelic or triallelic loci) was that of [47]. It was followed by a frequentist method supporting data on up to ten multiallelic loci [77], later incorporated into an R package called malaria.em; a frequentist method called MalHaploFreq, accounting for imperfect detection of minority clones [78]; a Bayesian method accounting for genotyping errors with a fixed miscall rate [79]; a frequentist method designed principally for prevalence estimation [80]; and a Bayesian method first described in [81] and later incorporated into an R package called FreqEstimationModel. All of these methods and more are described in detail in chapters two and three of [82]. A study comparing malaria.em, MalHaploFreq and FreqEstimationModel to two related methods was published in 2016 [5]. It put particular emphasis on limits of detection. An almost identical study followed, adding another related approach, but no associated software [6]. In 2022, a frequentist method called MultiLociBiallelicModel was published [62]. SNP-Slice [3] can also be viewed as a PLSF estimation method: PLSFs can be estimated from the output assignment matrix.

***Contemporary software*** PLSF estimates can be obtained with relative ease using FreqEstimationModel, MultiLociBiallelicModel and SNP-Slice (Malhaplofreq runs only on Microsoft Windows, while malaria.em is no longer maintained—Table S2).

FreqEstimationModel [81, 82] jointly generates posterior density estimates of the PLSFs, per-infection sequence counts and per-infection MOIs using an MCMC algorithm to fit a Bayesian model to categorical data on biallelic SNPs. Sequences are modelled as 2^n^ categories, where n is some number of biallelic SNPs, limited to at-most seven in practice. Per-infection MOIs are modelled as random variables from either a zero-truncated Poisson, Negative binomial, or Gamma distribution (parameterized using a prior estimate of the population-average MOI), or from a uniform distribution. Per-infection sequence counts are distributed according to a multinomial distribution, parameterized by sequence frequencies, which are assumed to be Dirichlet-distributed a priori. The multinomial construction allows the same sequence to be carried by multiple clones within an infection, which likely occurs frequently when MOI is high. FreqEstimationModel assumes independence between clones and does not account for genotyping errors. Missing data are integrated out by recursive sampling. A thorough description of FreqEstimationModel can be found in the third Chapter of [82].

MultiLociBiallelicModel [62] jointly generates MLEs of PLSFs and the population-average MOI using an EM algorithm to fit a frequentist model to categorical data on biallelic SNPs. Confidence intervals around MLEs are generated using the parametric bootstrap. Per-infection MOIs are modelled using a zero-truncated Poisson distribution whose parameter is the population-average MOI. Like FreqEstimationModel, sequences are modelled as 2^n^ categories, where n is the number of biallelic SNPs, limiting MultiLociBiallelicModel to some moderate number of SNPs in practice. Sequence counts are distributed according to a multinomial distribution, whose probability vector is the vector of allelic-sequence frequencies, allowing the same sequence to be carried by multiple clones within an infection. MultiLociBiallelicModel assumes clones are independent, does not account for genotyping errors, and cannot handle missing data.

SNP-Slice [3] outputs a maximum a posteriori estimate of all the sequence-to-infection assignments, from which PLSFs estimates can be obtained by averaging over infections. It marks a paradigm shift in the estimation of PLSFs because the Bayesian nonparametric approach circumvents the curse of dimensionality that limits all other PLSF estimation methods to moderate loci counts. SNP-Slice accommodates an unlimited number of sequences by modelling the assignment of sequences to infections as an Indian buffet process: a finite number of sequences (dishes) from an infinitely large selection (the buffet) are assigned to a finite number of infections (customers) [83]; and by modelling alleles within sequences as independent Bernoulli draws (with prior probability equal to 0.5 when the data are categorical, or to the major allele read count fraction when read counts are available). The sequence-to-infection assignment probabilities are modelled using a stick-breaking construction: bits of an unbroken stick of unit length (total probability) are progressively broken off, generating a set of assignment probabilities [84]. As an aside, posterior assignment probabilities are PLSF estimates, but the software does not output them at present. Marginally, the SNP-Slice model assumes a priori that per-infection sequence counts are Poisson distributed with mean equal to the population-average MOI; per-sequence assignment probabilities are Beta distributed with mean approximately equal to the reciprocal number of sequences circulating in the population; and haplotype-to-infection assignments are independent Bernoulli draws. If SNP-Slice is fit to data on loci under selection (e.g., markers of drug resistance), as is typically the case in studies of PLSFs, sequences are liable to be shared by multiple clones within infections, i.e., the true MOI will likely exceed the unique-sequence count, which is interpreted as an MOI estimate in [3]**.**

### Infection-level phase and frequency estimation

***Overview*** Infection-level phase and frequency estimation methods generally aim to reconstruct the sequences of whole-chromosome haplotypes within infections and possibly clonal proportions; per-infection MOI estimates can be viewed as byproducts. The problem of reconstructing sequences that characterize individuals in genetically diverse mixtures is widespread (haplotype assembly in human genetics, characterizing tumour diversity in oncology, quasispecies spectrum reconstruction in viral genomics, species resolution in metagenomics). There are no general solutions owing to each scenario having its own set of challenges, however each scenario generally requires very informative per-infection data. Some experimental methods (e.g., single-cell and long-read sequencing) may someday render infection-level phase and frequency estimation obsolete. However, these experimental methods are not yet optimized nor accessible at scale.

***Historical context*** The first infection-level “haplotype-estimating algorithm” for malaria parasites was designed to help monitor vaccine escape [85]. It was shown to reliably estimate the most likely combination of 6-SNP haplotypes within polyclonal *P. falciparum* infections with MOIs of two or three, but relied heavily on an experimental protocol. Over a decade later, a Bayesian method designed to identify sets of haplotypes, their joint phylogeny, and within-infection frequencies using short-read WGS data, was published, along with its application to data on *Plasmodium falciparum* apicoplasts extracted from clinical infections [86]. Unfortunately, the method does not support data from the sexually recombining nuclear genome of malaria parasites, because it relies on a phylogenetic model that assumes variation among haplotypes results from mutation not recombination. A subsequent method called DEploid was followed shortly after by an enhanced method, DEploidIBD [13, 74].

***Contemporary software*** The Bayesian mixture model of pfmix estimates proportions of intra-host clones but does not reconstruct their sequences because loci are assumed independent. SNP-Slice estimates infection-level sequences by assigning population-level sequences to infections, but not intra-host clone proportions because assignments are categorical (it is geared primarily towards inference on the population-level). DEploid and DEploidIBD estimate both intra-host sequences and proportions. In addition, DEploidIBD estimates IBD profiles between clones. In the descriptions of all of the methods above, the word strain is used instead of clone. Clone here is synonymous with strain in the descriptions of pfmix, DEploid, and DEploidIBD (these methods are designed for WGS data). In the description of SNP-Slice, the interpretation of the word strain depends on the data: if SNP-Slice is fit to limited loci under selection, strain in the description of SNP-Slice agrees with strain here (Table 1); if SNP-Slice is fit to data on many neutral loci, strain in the description of SNP-Slice is synonymous with clone here.

DEploid and DEploidIBD are Bayesian methods designed to deconvolute polyclonal infections one-by-one [13, 74]. Both methods require biallelic read count data from high-coverage WGS (median sequencing depth > 20, preferably without prior selective whole-genome amplification), PLAFs, and a panel of reference haplotypes. As in pfmix, observed read counts are modelled as beta-binomially distributed random variables, whose expectations are WSAFs multiplied by read-depths. WSAFs are based on latent clone proportions and haplotypes and then error adjusted, using a fixed miscall error rate for reads. Logit-transformed proportions are modelled as normally distributed random variables. Some maximum number of clones is assumed a priori (five for DEploid, four for DEploidIBD). The posterior MOI and effective MOI estimates are then computed using the posterior clone proportions that are greater than 0.01. As per [87], haplotypes are modelled as imperfectly copied mosaics of haplotypes in a reference panel using a hidden Markov model (HMM). The HMM transitions between the haplotypes in the reference panel with a rate governed by inter-marker distance and a scaled rate of recombination, which is assumed uniform across the genome. Under the DEploid model, haplotypes and proportions are estimated jointly in one step. DEploidIBD employs a two-step approach: firstly, IBD profiles, haplotypes, and proportions are estimated jointly using the aforementioned observation model and a HMM of IBD partitions; secondly, haplotypes are updated using the HMM of haplotype mosaics. Under the IBD-partition HMM, an initial partition is drawn uniformly at random from those compatible with a given number of IBD clusters. The IBD cluster count is drawn from a binomial distribution parameterized by the probability that two clones are not IBD at a given locus. Subsequent IBD partitions are redrawn with probability equal to there having been a recombination event; otherwise, the preceding IBD partition is copied. Conditional on the partition, haplotype alleles are drawn proportional to the PLAFs. DEploid is preferable when some clones have equal proportions; DEploidIBD is preferable when proportions are unbalanced and genetically distinct clones are related. Neither cope well with entirely balanced or extremely imbalanced clone proportions.

### Relatedness estimation

***Overview*** Typically, malaria parasite relatedness estimation aims to estimate a genome-average measure of IBD between malaria parasite genotypes (whole-genome sequence or subsets thereof). Unless identity-by-state (IBS) is used to approximate IBD (a model-free approach, e.g., [88]), statistical inference is required because IBD states are hidden. When parasites reside within polyclonal infections that are genotyped using bulk data, statistical inference is further required because MOI, frequency and phase are obfuscated.

***Historical context*** Pairwise relatedness estimation for malaria parasites is relatively new: DEploidIBD [13] generates estimates for intra-infection genotypes; whereas IsoRelate [89], hmmIBD [90], paneljudge [44] and Dcifer [91] generate estimates for inter-infection genotypes. It is not as new for humans and other diploids: the structure of the single-population model of hmmIBD, which is very similar to that of isoRelate and identical to that of paneljudge, is almost identical to an earlier model of an inbreeding coefficient for a single diploid eukaryote [92]. Under that model, errors affect both chromosomes of the diploid in unison (whereas haploid malaria parasites accumulate errors independently). The diploid model builds on a foundational study where IBD was modelled continuously along the genome [93].

***Contemporary software*** Pfmix and moimix generate *F*_*WS*_ estimates, which can be viewed as infection-level inbreeding coefficients, and thus measures of IBD averaged over all intra-infection haploid genotypes [32]*.* MOIRE also generates per-infection estimates of relatedness averaged over all intra-infection clones. DEploidIBD generates individual estimates of pairwise relatedness for each intra-infection genotype pair; all other relatedness estimation methods generate estimates for inter-infection genotypes and are frequentist.

Both isoRelate [89] and hmmIBD [90] were designed to analyse WGS data and are based on HMMs, where hidden states are either IBD or not (hmmIBD), or IBD counts of zero, one or two (isoRelate). The HMMs transition between IBD states with a rate governed by a recombination map and a switch-rate parameter. Under hmmIBD, a uniform recombination map is computed internally: inter-marker distances in base pairs are multiplied by a recombination rate whose default value (specified internally but easy to override) corresponds to *P. falciparum* (7.4e-7 Morgans per base pair [94]). To run isoRelate, the user provides a recombination map computed externally (i.e., the user has more freedom). Conditional on the hidden state, latent alleles are drawn with probabilities proportional to PLAFs. Both isoRelate and hmmIBD tolerate loci with missing data by summing over probabilities of all possible observations at said loci, and both account for genotyping miscall errors for each haploid genotype individually using an error model that also accommodates de novo mutations. Neither method outputs measures of uncertainty.

IsoRelate [89] uses unphased biallelic SNP data and PLAFs to generate relatedness estimates (estimated using method-of-moments, as in [95]) defined in terms of the probabilities of one or two IBD alleles, and IBD segments (generated using the Viterbi algorithm), for genotypes between infections with MOIs of one or two. Under the assumption that all within-infection IBS loci are IBD, isoRelate can also be used for infections with MOIs greater than two (isoRelate does not make any assumptions on intra-infection relatedness for infections with MOIs of two).

hmmIBD [90] is restricted to monoclonal samples but can be used to estimate relatedness (using an EM-based algorithm) and IBD segments (using the Viterbi algorithm) between infections from populations with different input allele frequencies. hmmIBD supports data from both biallelic and multiallelic markers, which are treated as categorical random variables. As such, distances between latent alleles and observations for microhaplotype and microsatellite markers are not accounted for. The probability of any error does increase with the number of alternative alleles, however.

Paneljudge [44] is based exactly on the single-population HMM of hmmIBD. It was designed to evaluate relatedness estimation using sparse data. As such, IBD segment estimation, which requires dense data, is not implemented. Instead, paneljudge generates confidence intervals (using the parametric bootstrap) around MLEs of relatedness (obtained via optimizing the likelihood, represented numerically using the forward algorithm)—confidence intervals are recommended for sparse data applications. In the case of missing data, for each pair separately, loci with missing data should be dropped and distances between markers without missing data should be computed.

Dcifer [91] generates MLEs of relatedness for genotypes across polyclonal infections using data on multiallelic markers (modelled as categorical random variables). Instead of using a HMM, independence between loci is assumed, precluding IBD segment estimation. In addition, Dcifer assumes all intra-infection genotypes are unrelated and each genotype in one infection can be related to at most one other in the other infection. Like isoRelate, Dcifer assumes infections come from populations whose allele frequencies are the same. It supports loci with missing data but requires MOI estimates. Dcifer does not account for genotyping errors or mutations, but errors were introduced into the simulated data used to evaluate it. Using the likelihood ratio approach, Dcifer provides measures of uncertainty with reliable coverage, and test statistics that can be used to separate unrelated and related infection pairs before generating more granular relatedness estimates (one relatedness estimate for each pair of inter-infection genetically distinct but related clones). It also returns two summary statistics: an estimate of the number of inter-infection genetically distinct but related clones and the sum of relatedness over all inter-infection genetically distinct but related clones (total relatedness). Compared with pairwise relatedness, total relatedness is easier to estimate and remarkably robust (even in the presence of simulated intra-infection relatedness). Depending on the target of inference, Newton’s method over either a one or higher dimensional grid is used to compute the likelihood and find MLEs.

### Outstanding challenges

The ultimate MrsFreqPhase method would estimate jointly all MrsFreqPhase targets of inference and more (e.g., population assignment, spatial spread). However, fully joint inference is infeasible at present: it would possibly entail a model of ancestral recombination [96]. More modest models are under development. Three notable gaps are as follows.

Given the increasing popularity of amplicon sequencing, there is a need for a PLSF estimation method that can exploit read counts on multiallelic loci as in [97], while circumventing the curse of dimensionality as in [3]. Ideally, the method would also support missing data, model genotyping errors, and account for imperfect detection [22, 65].

Also inspired by the increasing popularity of amplicon sequencing, there is a need for more sophisticated observation models that capture different error-structures for different marker types. For example, the observation {1,0,0} of three binary states is more similar to the latent microhaplotype {0,0,0} than the observation {1,1,0}. However, both are considered equally wrong when microhaplotype markers are modelled as categorical random variables—a common treatment because current allele-calling pipelines are analytically siloed from downstream analysis tools. Observation models could be integrated into MrsFreqPhase methods in a modular way, allowing the user to select the appropriate observation model for their data type, and ensuring a single method supports multiple data types. Stand-apart observation models can be used to test the sensitivity of estimates generated by MrsFreqPhase methods that lack adequate observation models: generate many observation-modified versions of an empirical data set under an appropriate stand-apart observation model; generate estimates for all data sets; if the estimates based on observation-modified datasets lead to a different conclusion, the conclusion is error-sensitive; otherwise, it is robust.

There are no methods capable of jointly estimating relatedness between genotypes both within and between infections (polyclonal infections can be deconvoled using DeploidIBD and then relatedness between statistically phased genotypes estimated using an inter-infection method). This is relevant because parasite infections are liable to contain related parasites across transmission settings (e.g., [11]). It could simplify the two-step procedure proposed above and, based on sensitivity analyses of the Dcifer method, it could improve the accuracy of relatedness estimation in low transmission intensity settings (in high transmission intensity settings, the relative contribution of related parasites is diminished by superinfection, limiting the return on investment). The latent state space of the model needs to account for all combinations of IBD states between genotypes both within and between infections. Inference is likely to be very challenging (the HMM of the first step of DEploidIBD, whose hidden state space includes combinations of IBD states between intra-infection genotypes alone, already struggles with four genotypes at most). That said, assuming loci-independence a priori (as in SNP-Slice) or entirely (as in MOIRE) could help.

## Conclusion

This review features many statistical genetic methods, some pre-dating the genomic era, designed specifically to estimate malaria parasite MOI, relatedness, frequency, and phase (MrsFreqPhase). The number of specialized methods and the maturity of the field is a testament to the extent to which studies of malaria depend on MrsFreqPhase methods.

That dependence is partly because methods from other fields cannot be repurposed easily due to the specificities of the malaria parasite life cycle. For example, methods used in viral genomic epidemiology are inappropriate because malaria parasites sexually recombine; meanwhile, methods used to analyse humans and other diploids are inappropriate because haploid malaria parasites reside in infections with unknown and potentially high MOIs. That is to say, a malaria infection is a bit like a polyploid eukaryote whose ploidy is variable and unknown.

Despite the maturity of the field, the malaria statistical genetic tool box is far from complete. Ultimately, MrsFreqPhase methods might converge under a joint inferential framework, with some features rendered obsolete by long-range or single-cell sequencing. This is a long way off, however: fully joint inference is extremely challenging while long-range and single-cell sequencing are not yet optimized, let alone accessible at scale. In the immediate term, MrsFreqPhase methods need to catch up with the growing popularity of amplicon sequencing, leveraging read count data from diverse multiallelic loci. Meanwhile, efforts are underway to harmonize the valuable but fragmented landscape of existing analysis tools through improved documentation, additional benchmarking (building on existing work by e.g., [3–6], and implementation of data and interoperability standards, with the ultimate goal of building a collaborative, transparent, and open platform of computationally interoperable software. That said, disjoint software, however interoperable, will never apply equally across the many diverse malaria epidemiologies of malaria; for that, a fully joint model is needed: a model of genetic selfing, inbreeding, and outcrossing, parasite brood and non-brood mating, host-to-host co-infection and superinfection, and all in the context of population dynamics and evolution (no small task; see [96]). And so, for the foreseeable future, users must stay abreast of the statistical basis of MrsFreqPhase methods in order to interpret results soundly and on a case-by-case basis.

## Supplementary Information

Additional file 1.

## Data Availability

No datasets were generated or analysed during the current study.

## Abbreviations

COI: Complexity of infection
FOI: Force of infection
IBD: Identity-by-descent
IBS: Identity-by-state
IDM: Imperfect-detection model
MCMC: Markov chain Monte Carlo
MLE: Maximum likelihood estimate
MOI: Multiplicity of infection
PLAF: Population-level allele frequency
PLSF: Population-level sequence frequency
SNP: Single-nucleotide polymorphism
WSAF: Within-sample allele frequency
WGS: Whole genome sequencing
