# Supplementary material for: Review of MrsFreqPhase methods: methods designed to estimate statistically malaria parasite multiplicity of infection, relatedness, frequency and phase
Source: Malar J. 2024 Oct 15;23:308. doi: 10.1186/s12936-024-05119-2 (PMC11481338; doi:10.1186/s12936-024-05119-2)
Supplement: Supplementary file 1 — Additional file 1. [file 12936_2024_5119_MOESM1_ESM.docx]

| **Table S1: Links to MrsFreqPhase software in order of appearance** |
| --- |
| MLMOI <https://github.com/Maths-against-Malaria/MOI-Bias-correction.git>  <https://cran.r-project.org/web/packages/MLMOI/index.html> |
| IDM (see supplement of [65] for upcoming github link) |
| MOIRE <https://github.com/EPPIcenter/moire> |
| MultiLociBiallelicModel <https://github.com/Maths-against-Malaria/MultiLociBiallelicModel> |
| pfmix <https://github.com/cascobayesian/pfmix> |
| moimix <https://github.com/bahlolab/moimix> |
| THEREALMcCOIL <https://github.com/EPPIcenter/THEREALMcCOIL>  <https://github.com/OJWatson/McCOILR> |
| Coiaf <https://github.com/bailey-lab/coiaf> |
| SNP-Slice <https://github.com/nianqiaoju/snp-slice> |
| FreqEstimationModel <https://github.com/aimeertaylor/FreqEstimationModel> |
| DEploid and DEploidIBD <https://github.com/DEploid-dev/DEploid>  <https://github.com/DEploid-dev/DEploid-r> |
| isoRelate <https://github.com/bahlolab/isoRelate> |
| hmmIBD <https://github.com/glipsnort/hmmIBD> |
| Paneljudge <https://github.com/aimeertaylor/paneljudge> |
| Dcifer <https://github.com/EPPIcenter/dcifer> |

| **Table S2: Summary of PLSF (population-level sequence frequency) estimation methods** | | | | | |
| --- | --- | --- | --- | --- | --- |
|  | MalHaploFreq  (Hastings and Smith, 2008) | Malaria.em  (Li et al., 2007) | FreqEstimationModel  (Taylor et al., 2014) | MultiLociBiallelic Model  (Tsoungui Obama and Schneider, 2022) | SNP-Slice  (Ju et al. 2023) |
| Format | An executable that runs on Microsoft Windows only | R package available from the CRAN archive but no-longer maintained in the CRAN repository | R package hosted on Github | R scripts hosted on Github with a user manual provided in the supplementary material of [62]. | An executable R file |
| Type of estimate generated | MLEs | MLEs | Numerical approximation of the joint posterior | MLEs | Maximum *a posteriori* estimates |
| Inference algorithm | Hill climbing algorithm | EM algorithm | MCMC (Metropolis-Hastings within Gibbs) | EM algorithm | MCMC (Slice sampling) |
| Uncertainty measures | Confidence intervals based on the profile-likelihood approach | Confidence intervals based on an estimate of the asymptotic variance-covariance matrix | Credible intervals based on percentiles of the numerical approximation | Confidence intervals based on the parametric bootstrap | Not generated / reported |
| Requires / generates per-infection MOI estimates | Requires | Per-infection MOI estimates can be generated *a posteriori* using population-level MLEs | Generates posterior density estimates directly | Per-infection MOI estimates can be generated *a posteriori* using population-level MLEs | Maximum *a posteriori* estimates of the MOI lower bound can be obtained by summing over per-infection sequence assignments |
| Practical limit on the number of loci | Three | Unstated but up to ten illustrated | Seven | Unstated but up to ten illustrated | Unlimited |
| Supports multiallelic as well as biallelic loci? | No | Yes | No | No | No |
| Supports read count data as well as categorical data? | No | No | No | No | Yes |
| Supports missing data (e.g., NAs)? | No | Yes | Yes | No | Yes |
| Models undetected clones? | Yes | No | No | No | No |
| Models genotyping errors? | No | No | No | No | Yes |
